# Silymarin Exerts Potent Antipruritic Effects Against Chloroquine-Induced Scratching in Mice by Modulating the NO/NF-κB/TRPA1 Pathway

**DOI:** 10.5812/ijpr-169711

**Published:** 2026-07-04

**Authors:** Hosna Bagheri, Amir Kiani, Mohammad Amin Ali Mohammad Golesorkhi, Antoni Sureda, Samira Shirooie

**Affiliations:** 1 Student Research Committee, Faculty of Pharmacy, Kermanshah University of Medical Sciences, Kermanshah, Iran; 2 Medical Biology Research Center, Health Technology Institute, Kermanshah University of Medical Sciences, Kermanshah, Iran; 3 Pharmaceutical Sciences Research Center, Health Institute, Kermanshah University of Medical Sciences, Kermanshah, Iran; 4 Research Group on Community Nutrition and Oxidative Stress (NUCOX) and Health Research Institute of Balearic Islands (IdISBa), University of Balearic Islands-IUNICS, Palma de Mallorca E-07122, Balearic Islands, Spain

**Keywords:** Silymarin, Chloroquine, Itching, Nitric Oxide, NF-κB P65, Pruritus

## Abstract

**Background:**

Pruritus may occur through histamine-dependent or histamine-independent mechanisms. Chronic pruritus, which is often unresponsive to antihistamines, has been associated with nitric oxide (NO) signaling, which can modulate nuclear factor-κB (NF-κB) activity and transient receptor potential ankyrin 1 (TRPA1) function.

**Objectives:**

This study investigated the antipruritic potential of silymarin in a mouse model of chloroquine-induced systemic pruritus.

**Methods:**

Adult male NMRI mice were allocated to 16 groups. Treatments included silymarin (10, 30, and 60 mg/kg), L-NAME (1 and 10 mg/kg), aminoguanidine, L-arginine (10 and 100 mg/kg), sildenafil (10 and 20 mg/kg), and various combinations of these agents with silymarin. Scratching behavior, including frequency and duration, was recorded for 30 minutes on the second day after chloroquine (CQ) administration. Dorsal root ganglia (DRG) were analyzed by immunofluorescence to assess the expression of NF-κB and TRPA1.

**Results:**

Silymarin (60 mg/kg), L-NAME (10 mg/kg), and aminoguanidine significantly reduced chloroquine-induced itching, whereas L-arginine (100 mg/kg) and sildenafil (20 mg/kg) exacerbated it. The combination of subeffective doses of L-NAME (1 mg/kg) and silymarin (10 mg/kg) produced potentiated antipruritic effects, whereas L-arginine and sildenafil counteracted the inhibitory effects of high-dose silymarin. CQ increased NF-κB and TRPA1 expression in the DRG. These changes were ameliorated by silymarin and L-NAME but aggravated by L-arginine and sildenafil.

**Conclusions:**

Silymarin alleviated chloroquine-induced itching, suggesting modulation of the NO/NF-κB/TRPA1 signaling pathway and supporting its potential as a therapeutic option for histamine-independent pruritic conditions.

## 1. Background

Pruritus, commonly referred to as itch, is defined as an unpleasant skin sensation that induces scratching behavior. When it persists for more than 6 weeks, it is classified as chronic pruritus (1). Chronic pruritus affects 8% to 25% of the population, for example, in Europe (2). At the molecular level, chronic pruritus involves pathways regulated by transient receptor potential cation channel subfamily V member 1 (TRPV1) and transient receptor potential ankyrin 1 (TRPA1) channels; T helper 2 cytokines, such as interleukin 31 (IL-31), that activate Janus kinases (JAKs) and signal transducer and activator of transcription proteins (STATs); and neuroimmune mediators (3). Itch transmission occurs through both histaminergic and nonhistaminergic C-fibers, with chronic itch predominantly mediated by the latter and often refractory to antihistamines (3). Chronic pruritus significantly impairs quality of life by causing neural sensitization, sleep disruption, mood disorders, and reduced daily functioning (1, 3).

To better understand these mechanisms and develop therapeutic approaches, various experimental models have been established to induce pruritus in animals and humans, including histaminergic methods such as intradermal histamine injection (4) and nonhistaminergic chemical stimuli such as cowhage, chloroquine, bovine adrenal medulla peptide 8 - 22 (BAM8 - 22), β-alanine, and cinnamaldehyde (4, 5). Other models include mechanical stimulation, such as light touch or brushing to assess alloknesis (4-6), electrical stimulation, thermal triggers, and visual cues, including contagious itch (6). These models enable differentiation of pruritic pathways and are widely used in both basic and clinical research.

Chloroquine (CQ) has been widely used to treat rheumatoid arthritis, systemic lupus erythematosus, and malaria. However, pruritus is a frequent adverse effect of CQ therapy, often leading to poor patient adherence and contributing to complications such as the emergence of CQ-resistant *Plasmodium falciparum*. Both acute intradermal and subcutaneous administration of CQ induce scratching behavior in rodents and humans (7). Mechanistically, CQ triggers pruritus primarily through activation of Mas-related G protein-coupled receptor A3 (MrgprA3) and its downstream effector, the TRPA1 ion channel (8, 9). Activation of MrgprA3 elevates intracellular calcium levels, which stimulates neuronal nitric oxide synthase (nNOS) and increases NO production (8). Recent evidence also indicates that CQ promotes reactive oxygen species (ROS) generation and intracellular alkalization, both of which can directly activate or sensitize TRPA1 and TRPV1 ion channels independently of MrgprA3 signaling (10).

TRPA1 is therefore recognized as a key ion channel mediating histamine-independent itch signaling. It is predominantly expressed in a subset of sensory neurons and is activated by various pruritogens, including chloroquine and other chemical stimuli (11, 12). TRPA1 activation leads to neuronal excitation and is essential for transmitting nonhistaminergic itch sensations (13). Moreover, NO modulates transient receptor potential (TRP) channels, such as TRPA1 and TRPV1, via cysteine S-nitrosylation, thereby facilitating neuronal depolarization and signal transmission (14). In addition, the Gβγ subunit released following MrgprA3 activation may activate adenylyl cyclase pathways, further increasing intracellular calcium levels and nNOS activity (9). These findings highlight TRPA1 as a central player in both CQ-dependent and CQ-independent itch mechanisms and a potential therapeutic target (15).

The canonical NO-cyclic guanosine monophosphate (cGMP)-protein kinase G (PKG) signaling pathway includes NO synthesis by nNOS, activation of soluble guanylyl cyclase, elevation of cGMP, and subsequent PKG activation. This pathway regulates NF-κB, a transcription factor implicated in inflammatory and neuroimmune responses related to pruritus (16). Phosphorylation of the NF-κB inhibitor IκB, mainly by IκB kinase and also by PKG, promotes NF-κB translocation into the nucleus. In the nucleus, NF-κB promotes transcription of proinflammatory cytokines such as interleukin 1β (IL-1β), interleukin 6 (IL-6), and tumor necrosis factor α (TNF-α), as well as proangiogenic factors, including vascular endothelial growth factor, matrix metalloproteinase 2 (MMP-2), and MMP-9 (17, 18).

The functional relevance of this pathway has been demonstrated pharmacologically. In this study, L-NAME, a nonselective nitric oxide synthase (NOS) inhibitor, and aminoguanidine, a selective nNOS inhibitor, were used as negative controls because they significantly reduce CQ-induced scratching. Conversely, L-arginine, an NO precursor, and sildenafil, a phosphodiesterase 5 inhibitor, served as positive controls that enhance scratching behavior, thereby inferring the involvement of the NO/cGMP pathway through pharmacological modulation without direct measurement of NO, nitrite, cGMP, or PKG levels (19). Clarifying these molecular mechanisms and their pharmacological modulators may provide a rationale for developing targeted therapies to alleviate CQ-induced pruritus.

Given the involvement of oxidative stress and inflammatory pathways in CQ-induced pruritus, natural compounds with antioxidant and anti-inflammatory properties are of particular interest. Among them, silymarin, a complex mixture of flavonolignans derived from the seeds of the milk thistle plant (*Silybum marianum*), has attracted increasing attention for its therapeutic potential, particularly in liver diseases (20). Its main active components include silybin, isosilybin, silydianin, and silychristin, with silybin being the most potent and extensively studied compound (21). Silymarin exhibits several pharmacological properties, including antioxidant, anti-inflammatory, and antifibrotic activities, which are largely attributed to its ability to scavenge free radicals, modulate immune responses, and inhibit the production of proinflammatory cytokines (22). Beyond hepatic effects, silymarin has been shown to exert protective effects against lung injury by lowering NO production, limiting inflammatory cell infiltration and myeloperoxidase activity, and restoring antioxidant defenses such as catalase, superoxide dismutase, and glutathione (23-25). In addition, supplementation with silymarin may increase interleukin 10 levels, potentially through suppression of the NF-κB proinflammatory signaling cascade, thereby enhancing anti-inflammatory signaling (26-28).

## 2. Objectives

Building on this rationale, the present study investigated the contribution of the NO/cGMP/PKG signaling pathway and TRPA1 ion channel activity in a mouse model of CQ-induced pruritus, while also evaluating the anti-inflammatory and antipruritic effects of silymarin, a natural antioxidant. Elucidation of these pathways may provide valuable insights for developing targeted and more effective therapies for chronic drug- and disease-associated pruritus.

## 3. Methods

### 3.1. Chemicals

Milk thistle extract (*Silybum marianum*; > 80% silymarin, > 30% silybin) was purchased from Gol Daroo Company (Isfahan, Iran) [Catalog No., Batch No.] and dissolved in olive oil. Chloroquine was purchased from Pars Daroo, and sildenafil was obtained from Actover Company. L-Nitroarginine methyl ester (L-NAME), aminoguanidine (AG), and L-arginine (L-Arg) were purchased from Sigma-Aldrich (Buchs, Switzerland) and prepared according to the manufacturers’ instructions. Polyclonal primary antibodies against mouse TRPA1 (ab-58844) and NF-κB p65 (ab-16502) were used for immunofluorescence assays. Secondary antibodies were goat anti-rabbit IgG H&L conjugated with Alexa Fluor 488 (ab150077) and Alexa Fluor 594 (ab150080), diluted in 10% normal goat serum to reduce nonspecific binding. All compounds were administered intraperitoneally at the doses indicated in Table 1.

**Table 1.** Injection and Gavage Procedures Used in the Study Groups ^a^

Groups	Subcutaneous Injection, 400 μL/site	Intraperitoneal Injection, 30 min Before CQ	Gavage of Silymarin Dissolved in Olive Oil, 4 h Before CQ
**Group 1 (control)**	Distilled water	-	Olive oil only
**Group 2**	CQ 400 μL/site	-	-
**Group 3**	CQ 400 μL/site	-	Silymarin 10 mg/kg
**Group 4**	CQ 400 μL/site	-	Silymarin 30 mg/kg
**Group 5**	CQ 400 μL/site	-	Silymarin 60 mg/kg
**Group 6**	CQ 400 μL/site	L-NAME 1 mg/kg i.p.	-
**Group 7**	CQ 400 μL/site	L-NAME 10 mg/kg i.p.	-
**Group 8**	CQ 400 μL/site	AG 200 mg/kg i.p.	-
**Group 9**	CQ 400 μL/site	L-ARG 10 mg/kg i.p.	-
**Group 10**	CQ 400 μL/site	L-ARG 100 mg/kg i.p.	-
**Group 11**	CQ 400 μL/site	Sild 10 mg/kg i.p.	-
**Group 12**	CQ 400 μL/site	Sild 20 mg/kg i.p.	-
**Group 13**	CQ 400 μL/site	L-NAME 1 mg/kg i.p.	Silymarin 10 mg/kg
**Group 14**	CQ 400 μL/site	AG 200 mg/kg i.p.	Silymarin 60 mg/kg
**Group 15**	CQ 400 μL/site	L-ARG 100 mg/kg i.p.	Silymarin 10 mg/kg
**Group 16**	CQ 400 μL/site	Sild 20 mg/kg i.p.	Silymarin 60 mg/kg

^a^ Abbreviations: AG, aminoguanidine; CQ, chloroquine; i.p., intraperitoneal; L-ARG, L-arginine; L-NAME, nitro-L-arginine methyl ester; Sild, sildenafil.

### 3.2. Animals

A total of 128 male NMRI mice, aged (6 - 8) weeks and weighing 25 ± 2 g, were purchased from Elm Bavaran Aftab Company (Iran). Upon arrival, the animals were acclimated for 7 days before any experimental procedures. Mice were group-housed, with 2 to 4 animals per standard polycarbonate cage, with standard wood-shaving bedding and environmental enrichment items. They were maintained under identical laboratory conditions, including a 12:12-hour light/dark cycle, a constant temperature of 25°C, and ad libitum access to standard rodent chow and water. All animals were healthy. During behavioral testing, mice were evaluated individually in observation chambers to minimize stress. Animals were randomly assigned to experimental groups (n = 8 per group) using a computer-based random number generator (GraphPad Prism), ensuring equal allocation probability and eliminating selection bias (26).

### 3.3. Animal Grouping and Itch Induction

Sixteen groups of male mice (n = 8 per group) were randomly assigned as described in Table 1. Based on previous studies using chloroquine-induced scratching models and our preliminary experiments, we selected n = 8 mice per group, which provides sufficient power (> 80%) to detect moderate-to-large differences in scratching behavior and molecular endpoints while minimizing animal use. One day before treatment, the dorsal skin of each mouse was shaved using a depilatory cream. Pruritus was induced by subcutaneous injection of 400 μg/site of chloroquine into the shaved area. To ensure allocation concealment, an independent researcher prepared the treatment solutions in identical vials labeled with neutral codes. Accordingly, the investigators responsible for administering the treatments and inducing itch were blinded to group assignments until the end of the experimental phase.

The injection and gavage procedures for each group are summarized in Table 1.

### 3.4. Behavioral Assessment of Scratching

The experimental protocol was conducted over 2 consecutive days. On both days, interventions were administered as a single daily dose before chloroquine injection. Silymarin was administered orally 4 hours before CQ to coincide with its peak plasma concentration (27). NO-pathway modulators were injected intraperitoneally 30 minutes before CQ, reflecting their rapid systemic distribution (28).

Immediately after CQ injection, scratching behavior was evaluated in all animals during a 30-minute observation period on the second day of treatment in a quiet environment with minimal disturbance. Both scratching frequency and duration were recorded by direct visual observation by an experimenter blinded to the treatment groups. Scratching duration was measured in seconds using a stopwatch. Scratching frequency was defined as the number of distinct scratching bouts, with a bout considered distinct if scratching movements occurred at a new anatomical site or were separated from the previous bout by a pause (29). Immediately after the 30-minute behavioral assessment on day 2, the same animals were anesthetized and euthanized for DRG tissue collection, ensuring a direct correlation between behavioral phenotypes and immunofluorescence data.

Behavioral observations were conducted by an investigator blinded to the treatment protocols, ensuring that the scoring of scratching frequency remained unbiased and independent of the experimental groups.

### 3.5. Tissue Collection

On the second day, after behavioral assessments, mice were deeply anesthetized with an intraperitoneal injection of ketamine (90 mg/kg) and xylazine (10 mg/kg). Following blood collection, the DRG of the spinal cord were carefully dissected under a stereomicroscope and fixed in 10% neutral-buffered formalin for 72 hours. Fixed tissues were then processed for immunofluorescence staining according to standard protocols (30).

### 3.6. TRPA1 and NF-κB p65 Immunostaining

Immunofluorescence staining was performed to evaluate NF-κB and TRPA1 expression. Formalin-fixed DRG samples were sectioned into 5-μm slices and mounted on poly-L-lysine-coated slides. Sections were deparaffinized, rehydrated through a graded ethanol series, and rinsed with distilled water. After 2 washes with TBS containing 0.1% Triton X-100 (2 × 5 min), antigen retrieval was performed in citrate buffer (pH 6.0) by microwave heating at 700 W for 10 minutes. After cooling, nonspecific binding sites were blocked with 10% normal donkey serum in TBS for 2 hours at room temperature. Samples were then incubated with primary antibodies (rabbit polyclonal anti-NF-κB p65 [1:100 dilution, Abcam, USA] or rabbit polyclonal anti-TRPA1 [1:100 dilution, Abcam, USA]) using the Turbo protocol, a rapid incubation method to improve antibody binding, with initial incubation at 37°C for 4 hours followed by overnight incubation at 4°C.

After three 5-minute washes, slides were incubated with secondary antibodies (donkey anti-rabbit Alexa Fluor 488, 1:200, Invitrogen, USA) diluted in 10% normal donkey serum for 2 hours at room temperature, followed by overnight incubation at 4°C. After washing and counterstaining with DAPI (1 μg/mL; Sigma-D9542) for 15 minutes at room temperature, slides were mounted with anti-fade glycerol/PBS mounting medium. Fluorescence images were captured using an Olympus fluorescence microscope, and TRPA1 and NF-κB expression levels were quantified using ImageJ software by measuring mean fluorescence intensity in randomly selected fields. Photographs were taken with an Olympus BX50 fluorescence microscope and an Olympus DP72 camera (31).

To maintain blinding during molecular outcome assessment, all acquired images were assigned unique alphanumeric codes by an independent researcher. This ensured that the investigator performing fluorescence intensity quantification for TRPA1 and NF-κB remained unaware of the experimental groups. The original group identities were re-linked to the coded data only after completion of the entire quantification and statistical analysis process.

### 3.7. Statistical Analysis

Statistical analyses were performed using GraphPad Prism version 9. Data are presented as mean ± standard error of the mean (SEM). Normality of data distribution was assessed using the Shapiro-Wilk test, and homogeneity of variances was evaluated using Levene’s test. One-way analysis of variance (ANOVA) followed by Tukey’s post hoc test and two-way ANOVA followed by Bonferroni’s post hoc test were used to compare differences between groups. A P value of less than 0.05 was considered statistically significant.

Although a formal a priori sample size calculation was not strictly performed, the allocation of n = 8 mice per group was justified based on well-established protocols for chloroquine-induced itch models (26). Based on historical variance estimates from these models, this sample size yields a statistical power of approximately 80% (α = 0.05) to detect meaningful differences in our primary endpoints: scratching frequency and scratching duration. Furthermore, this study design was adequately powered to evaluate both the individual dose-response effects of silymarin and the interaction effects between silymarin and NO modulators.

Statistical analysis was performed by a blinded analyst using the coded datasets. Experimental group identities were unveiled only after completion of the final statistical results and comparisons.

## 4. Results

### 4.1. Behavioral Results

Behavioral assessments were performed to evaluate the effects of chloroquine and the various treatment groups on scratching behavior, including scratching frequency and duration.

### 4.2. Effects of Individual Treatments on Scratching Frequency and Duration

**Figure 1.**
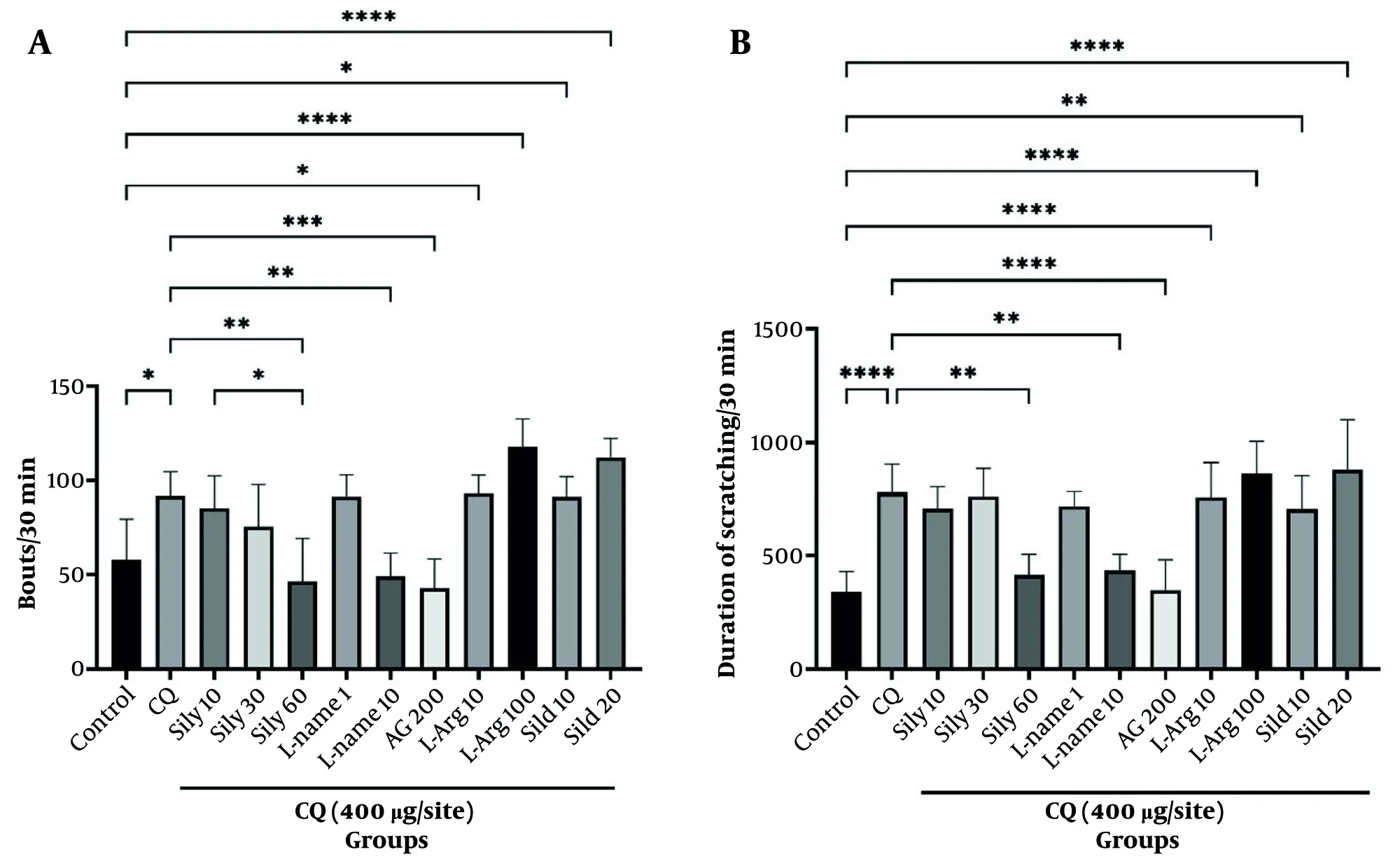
Effects of silymarin (Sily) on chloroquine (CQ)-induced scratching in mice. Panel A shows scratching frequency, and Panel B shows scratching duration. Abbreviations: AG, aminoguanidine; L-Arg, L-arginine; Sild, sildenafil. Values are shown as mean ± SEM (n = 8). * P < 0.05, ** P < 0.01, *** P < 0.001, **** P < 0.0001.

Subcutaneous injection of chloroquine significantly increased both scratching frequency (P < 0.05; Figure 1A) and duration (P < 0.0001; Figure 1B) compared with the control group. Co-administration of silymarin at 10 or 30 mg/kg with CQ slightly reduced scratching frequency and duration; however, these reductions did not reach statistical significance. In contrast, silymarin at 60 mg/kg combined with CQ significantly decreased both the number of scratching bouts (P < 0.01; Figure 1A) and total scratching time (P < 0.01; Figure 1B). L-NAME at 10 mg/kg effectively attenuated CQ-induced scratching, reducing frequency (P < 0.01; Figure 1A) and duration (P < 0.01; Figure 1B), whereas L-NAME at 1 mg/kg had no significant effect. Aminoguanidine at 200 mg/kg also significantly decreased frequency (P < 0.01; Figure 1A) and duration (P < 0.001; Figure 1B). L-arginine at 10 mg/kg significantly increased frequency (P < 0.05; Figure 1A) and duration (P < 0.0001; Figure 1B), and L-arginine at 100 mg/kg produced similar effects (Figure 1A and 1B). Sildenafil at 10 mg/kg increased both frequency (P < 0.05; Figure 1A) and duration (P < 0.0001; Figure 1B), and sildenafil at 20 mg/kg also increased frequency (P < 0.05; Figure 1A) and duration (P < 0.0001; Figure 1B).

### 4.3. Co-Administration of Silymarin With NOS Modulators: Synergistic and Antagonistic Effects

**Figure 2.**
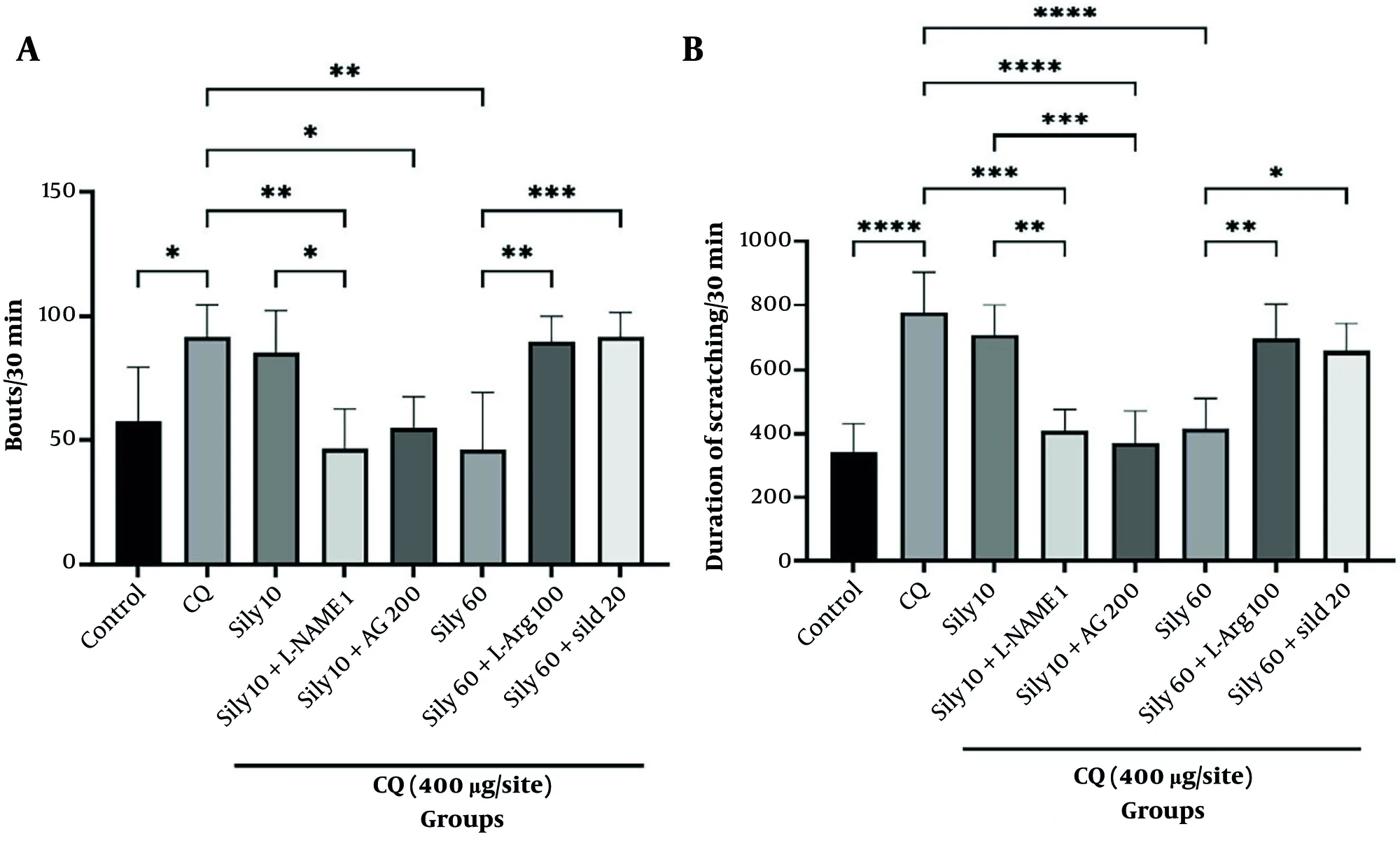
Effects of silymarin (Sily) on chloroquine (CQ)-induced scratching in mice. Abbreviations: AG, aminoguanidine; L-Arg, L-arginine; Sild, sildenafil. Panel A shows scratching frequency, and Panel B shows scratching duration. Values are shown as mean ± SEM (n = 8). * P < 0.05, ** P < 0.01, *** P < 0.001, **** P < 0.0001.

Mice receiving silymarin 10 mg/kg combined with L-NAME 1 mg/kg exhibited a significant reduction in both scratching frequency (P < 0.05; Figure 2A) and duration (P < 0.01; Figure 2B) compared with mice treated with silymarin 10 mg/kg, indicating a synergistic effect at a dose of silymarin that was otherwise ineffective. Similarly, silymarin 10 mg/kg combined with aminoguanidine 200 mg/kg significantly decreased scratching frequency (P < 0.05; Figure 2A) and duration (P < 0.0001; Figure 2B) compared with CQ plus silymarin 10 mg/kg.

Conversely, mice treated with silymarin 60 mg/kg plus L-arginine 100 mg/kg showed a significant increase in scratching frequency (P < 0.01; Figure 2A) and duration (P < 0.01; Figure 2B) compared with those treated with silymarin 60 mg/kg, indicating that L-arginine reversed the antipruritic effect of silymarin at an effective dose. Likewise, co-administration of silymarin 60 mg/kg plus sildenafil 20 mg/kg significantly increased both scratching frequency (P < 0.001; Figure 2A) and duration (P < 0.05; Figure 2B) relative to silymarin 60 mg/kg, confirming an antagonistic interaction.

### 4.4. Effects of Silymarin on TRPA1 and p65 NF-κB Protein Expression

**Figure 3.**
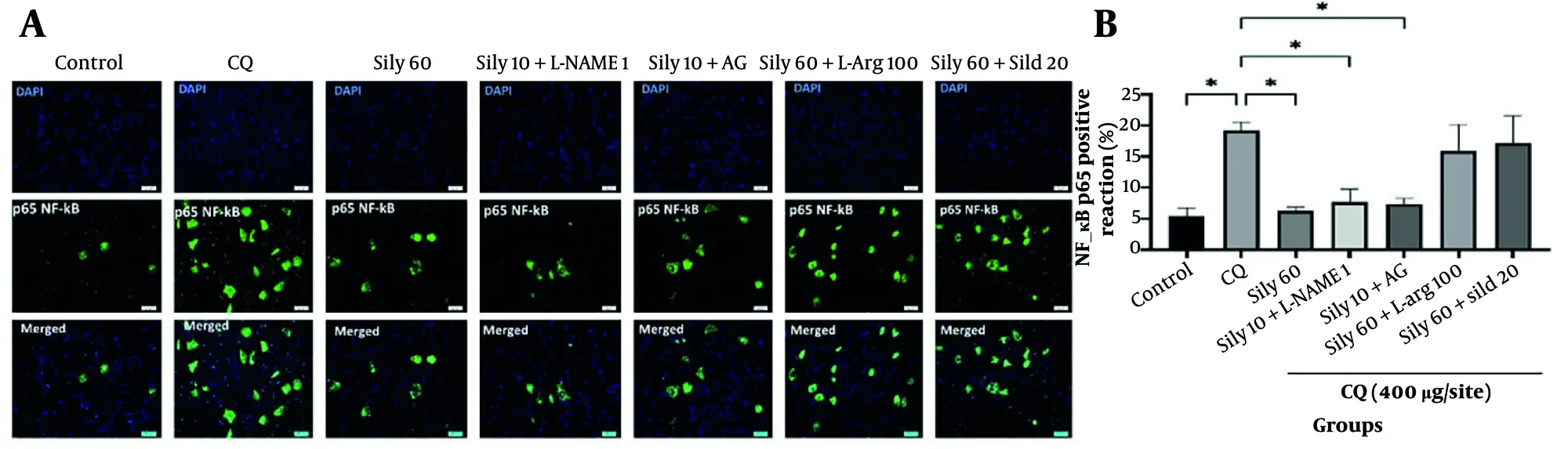
p65 NF-κB expression in mouse spinal dorsal root ganglia (DRG) evaluated by fluorescence immunohistochemistry. Abbreviations: AG, aminoguanidine; CQ, chloroquine; L-Arg, L-arginine; Sild, sildenafil; Sily, silymarin. A, shows representative immunofluorescence images, and B, shows optical density quantification of the immunofluorescence signal using ImageJ. Data are shown as mean ± SEM. * P < 0.05.

**Figure 4.**
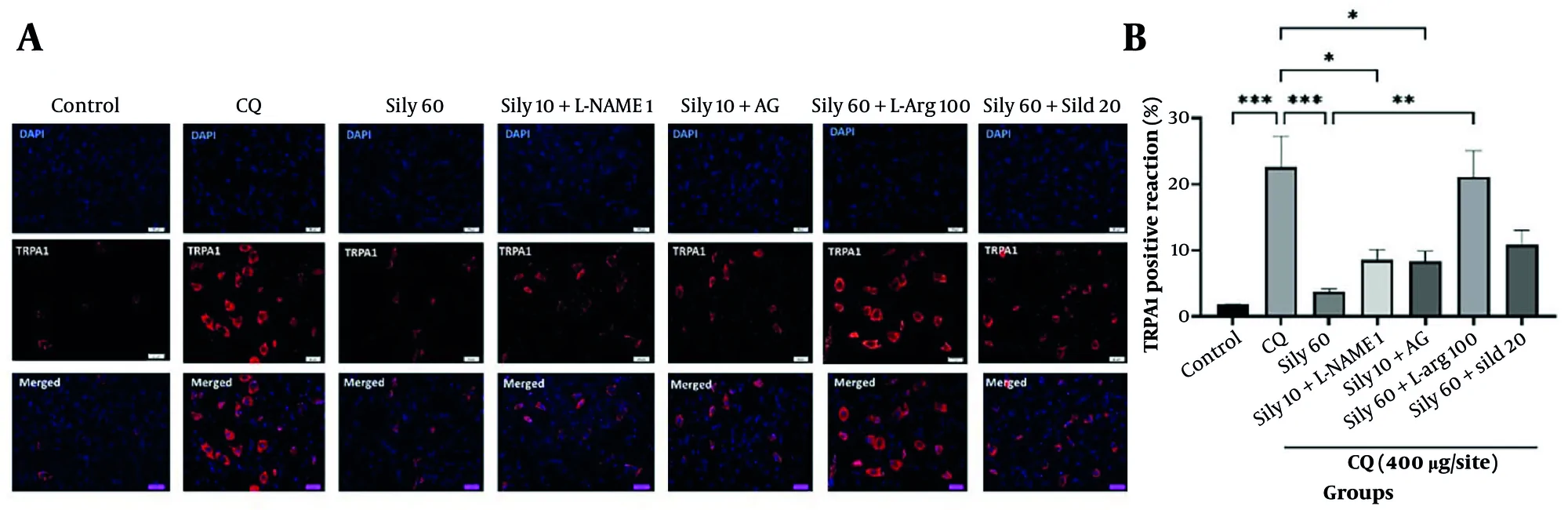
TRPA1 expression in mouse spinal dorsal root ganglia (DRG) evaluated by fluorescence immunohistochemistry. Abbreviations: AG, aminoguanidine; CQ, chloroquine; L-Arg, L-arginine; Sild, sildenafil; Sily, silymarin. A, shows representative immunofluorescence images, and B, shows optical density quantification of the immunofluorescence signal using ImageJ. Data are shown as mean ± SEM. * P < 0.05, ** P < 0.01, *** P < 0.001.

Immunofluorescence analysis of NF-κB and TRPA1 protein expression is shown in Figures 3 and 4. In the CQ group, expression levels of p65 NF-κB and TRPA1 were significantly higher than those in the control group (P < 0.05 and P < 0.001, respectively). Oral administration of silymarin (60 mg/kg) significantly reduced the elevated p65 NF-κB and TRPA1 expression levels observed in the CQ group (P < 0.05 and P < 0.001, respectively). The subeffective dose of silymarin (10 mg/kg) combined with subeffective doses of L-NAME (1 mg/kg) or aminoguanidine (200 mg/kg) significantly reduced p65 NF-κB and TRPA1 levels in the CQ group (P < 0.05). Figure 4 specifically presents the TRPA1 immunofluorescence findings and optical density quantification in mouse DRG.

## 5. Discussion

The present study demonstrates a pharmacological association between silymarin and NOS inhibitors, namely L-NAME and aminoguanidine, in attenuating chloroquine-induced scratching behavior in animal models. Co-administration of subeffective doses of silymarin with subeffective doses of either NOS inhibitor produced a marked additive reduction in CQ-induced scratching, suggesting that these agents may converge on a shared signaling mechanism, most plausibly the NO/cGMP/PKG cascade, to exert their antipruritic effects. The NO/cGMP/PKG signaling pathway is increasingly recognized as a critical mediator of itch signaling and a promising target for therapeutic intervention (32). Pharmacological modulation of NOS activity significantly alters itch-related behaviors, supporting the potential of NOS inhibitors in antipruritic therapies (33). In addition, silymarin exerts notable antioxidant and anti-inflammatory effects that may contribute to its protective role in skin disorders associated with itch (34). Conversely, L-arginine and sildenafil reversed the antipruritic effects of 60 mg/kg silymarin and concurrently increased TRPA1 expression, indicating a bidirectional modulatory influence on this pathway (35).

Furthermore, recent evidence has expanded our understanding of the role of NO in skin sensitization and inflammatory responses. NO functions as a signaling molecule that modulates both innate and adaptive immune responses by regulating immune cell activity and cytokine production, thereby contributing to skin sensitization and inflammation, which are key components of pruritus pathophysiology. This immunomodulatory role of NO is consistent with the antagonistic effects observed with L-arginine and sildenafil and underscores a pharmacological association with the NO/cGMP/PKG signaling pathway in the antipruritic actions of silymarin (36). Consistent with these observations, Wang et al. (35) reported that NO scavenging alleviates pruritus by inhibiting S-nitrosylation of TRP channels and reducing calcium influx in sensory neurons, further supporting NOS inhibition as an effective antipruritic strategy. These findings align with our data showing that NOS inhibitors potentiate silymarin-mediated suppression of CQ-induced scratching behavior (35). Co-administration of subeffective doses of silymarin with NOS modulators produced potentiating antipruritic effects, consistent with involvement of the NO/NF-κB/TRPA1 pathway. Importantly, NO-pathway involvement was inferred exclusively from pharmacological modulation rather than directly demonstrated by measurements of NO, nitrite, cGMP, or PKG.

Several studies indicate that the NO/cGMP/PKG pathway is subject to bidirectional regulation by both positive and negative modulators and that this regulation is crucial for sensory neuron sensitization and pruritus (3, 37-39). In our experiments, NOS inhibitors, including L-NAME and aminoguanidine, functioned as negative modulators and enhanced the antipruritic effect of silymarin. In contrast, L-arginine and sildenafil acted as positive modulators, reversed the effects of silymarin, and increased TRPA1 expression. In this context, our results are consistent with evidence that NO scavenging alleviates scar pruritus by inhibiting S-nitrosylation of TRP channels and limiting calcium influx in sensory neurons (35). However, the balance between opposing modulatory influences highlights the complexity of therapeutically targeting the NO pathway and emphasizes that both inhibition and enhancement of NO signaling can profoundly affect itch outcomes.

It is important to note that the NO/cGMP/PKG pathway is unlikely to be the only mechanism underlying the antipruritic action of silymarin. Silymarin is also recognized for its anti-inflammatory and antioxidant properties, including inhibition of NF-κB activation and reduction of ROS, both of which act as upstream regulators of TRPA1 expression and neuronal excitability. Specifically, multiple TRP channels, including TRPA1, TRPV1, and transient receptor potential melastatin 8 (TRPM8), participate in sensory neuron excitation by pruritogens such as CQ. Although our study focused primarily on TRPA1, investigating whether silymarin modulates other TRP channels will be necessary to fully characterize its therapeutic profile (40).

A recent review highlighted that silymarin exerts clinical anti-inflammatory effects by inhibiting NF-κB signaling, along with the mitogen-activated protein kinase and JAK-STAT3 pathways, with consequent regulation of inflammatory mediator secretion (41).

Previous work has extensively characterized positive and negative modulation of the NO/cGMP/PKG pathway using agents such as L-NAME, aminoguanidine, L-arginine, and sildenafil, particularly in the contexts of pain, inflammation, and neuronal sensitization. L-NAME and aminoguanidine reduce NO production and can attenuate inflammatory and neuropathic symptoms (19, 42). Conversely, L-arginine, a substrate for NOS, and sildenafil, a phosphodiesterase 5 inhibitor, increase cGMP levels and potentiate NO signaling, which can enhance neuronal excitability and sensitization (19). Although these modulatory effects are well documented in pain and inflammation models, our data extend their relevance to chloroquine-induced pruritus and demonstrate a previously underappreciated additive interaction between silymarin and NOS inhibition (19).

The behavioral findings of this study further substantiate the involvement of the NO/cGMP/PKG pathway in CQ-induced pruritus and its modulation by silymarin. The 60 mg/kg dose of silymarin significantly reduced both scratching frequency and duration, whereas lower doses were largely ineffective. Notably, NOS inhibitors alone produced marked antipruritic effects, and co-administration of a subeffective dose of silymarin (10 mg/kg) with L-NAME or aminoguanidine produced a significant potentiating reduction in scratching. These convergent behavioral outcomes reinforce the hypothesis that silymarin and NOS inhibitors act on a common pathway, likely the NO/cGMP/PKG cascade. Conversely, administration of L-arginine or sildenafil reversed the effect of silymarin and, when administered alone, exacerbated scratching, further supporting their facilitatory role. The consistency of these effects across assessments of scratching frequency and duration supports the robustness of the behavioral data and reinforces the mechanistic hypothesis proposed in this study.

In addition, immunofluorescence analysis of DRG tissues revealed significant upregulation of the inflammatory markers NF-κB p65 and TRPA1 in the CQ-treated group compared with the control group. Silymarin administered at 60 mg/kg significantly attenuated these increases (P < 0.05 and P < 0.001). Furthermore, co-treatment with subeffective doses of silymarin (10 mg/kg) and NOS inhibitors significantly reduced these markers compared with CQ alone (P < 0.05), indicating potentiation of anti-inflammatory effects. Conversely, silymarin co-administered with L-arginine or sildenafil reversed marker suppression, consistent with the observed behavioral antagonism.

Taken together, the molecular and behavioral data support a model in which silymarin alleviates chloroquine-induced pruritus in association with attenuated NO signaling, suppressed NF-κB-mediated inflammation, and downregulated TRPA1 expression. The observed additive effects with NOS inhibitors further emphasize the therapeutic relevance of targeting the NO/cGMP/PKG pathway in chronic pruritus and highlight the multifaceted antipruritic potential of silymarin through modulation of neuroinflammatory pathways and sensory ion channels.

This study provides several novel contributions to understanding the antipruritic actions of silymarin and its interaction with the NO signaling pathway. First, we demonstrate a significant potentiating interaction between silymarin and NOS inhibitors in reducing itch behavior, highlighting the central role of the NO/cGMP/PKG pathway in mediating antipruritic effects. Second, we integrate behavioral and molecular analyses of TRPA1 and NF-κB p65 to provide a multidimensional perspective on the mechanisms of action of silymarin. Third, we describe bidirectional regulation of the NO pathway by positive modulators, including L-arginine and sildenafil, and negative modulators, including L-NAME and aminoguanidine, clarifying how opposing influences on the same signaling axis can differentially modulate pruritus. These findings highlight the therapeutic promise of silymarin as a multifaceted agent for managing chronic pruritus and provide a basis for future studies to explore targeted interventions within NO signaling and inflammatory pathways.

### 5.1. Limitations

This study has several limitations that should be acknowledged when interpreting the results. First, the sample size was limited, which may reduce the generalizability of the findings to broader populations. Second, we did not identify the predominant cellular sources or NOS isoforms responsible for the observed effects, such as neuronal, inducible, or endothelial NOS; therefore, the relative contribution of each remains unclear. Third, NO-pathway involvement was inferred exclusively from pharmacological modulation, as direct measurements of NO, nitrite, cGMP, or PKG levels were not performed. Thus, more sensitive and continuous techniques, such as microdialysis, would provide more definitive information on NO dynamics. Fourth, the study focused on short-term effects, and the long-term efficacy of silymarin and NOS modulators was not assessed, which is crucial for clinical application. Finally, only a single itch model, the chloroquine-induced model, was used, limiting extrapolation to other etiologies of chronic pruritus in humans.

### 5.2. Future Directions

To extend these findings, future work should increase sample sizes and include both sexes to improve statistical robustness and assess sex-dependent effects. Detailed investigation of nitric oxide sources in both peripheral and central components of the itch pathway is warranted. Continuous, real-time assessment of NO and its downstream effectors, using methods such as microdialysis or electrochemical sensors, would enable direct correlation of these signaling dynamics with behavioral and molecular endpoints. Longitudinal studies are necessary to evaluate the long-term efficacy and safety of silymarin and NOS modulators in chronic pruritus models. In addition, exploring the effects of silymarin on other molecular pathways involved in itch, including inflammatory cascades and ion channels, would provide further insight. Finally, clinical trials are essential to determine the therapeutic potential and safety profile of silymarin in patients with chronic pruritus.

### 5.3. Conclusions

The results of this study suggest that the antipruritic effect of silymarin against nonhistaminergic chloroquine-induced itch is associated with modulation of the NO pathway, potentially via inhibition of NOS activity, together with suppression of NF-κB signaling, TRPA1 expression, and MMP activity. Agents that inhibit NO production reduced itch behavior, and co-administration of NOS inhibitors with silymarin produced additive antipruritic effects, whereas positive modulators of NO signaling reversed the benefits of silymarin. Because direct measurements of NO or cGMP were not performed, these findings should be interpreted as pharmacological associations consistent with involvement of the NO/NF-κB/TRPA1 pathway rather than definitive mechanistic causality. Given the mechanistic parallels between chloroquine-induced itch and other chronic pruritic disorders, targeting the NO signaling axis, potentially in combination with multitarget agents such as silymarin, represents a promising therapeutic strategy warranting further investigation. These preclinical findings support further evaluation of silymarin in clinical trials for histamine-independent chronic pruritus.

## Data Availability

The dataset presented in the study is available on request from the corresponding author during submission or after publication.
